# Prediction Models for In-Hospital Delirium Using Routinely Collected Electronic Health Record Data: Systematic Review

**DOI:** 10.2196/91618

**Published:** 2026-09-16

**Authors:** Hung-Min Huang, Chun-Shun Lu, Geng-Wei Chang, Ming-Hsu Tien, Yu-Kai Hsu

**Affiliations:** 1 Institute of Health Informatics, University College London, Gower Street, London, England, WC1E 6BT, United Kingdom, 44 7570909461; 2 Department of General Medicine, MacKay Memorial Hospital, Taipei, Taiwan; 3 Department of General Medicine, Chang Gung Memorial Hospital, Taipei, Taiwan; 4 Department of General Medicine, Far Eastern Memorial Hospital, Taipei, Taiwan

**Keywords:** delirium, risk prediction, electronic health records, machine learning, clinical decision support, inpatients, systematic review, PROBAST, TRIPOD

## Abstract

**Background:**

Delirium is a common and clinically important form of acute in-hospital mental status deterioration. Electronic health record (EHR)–based prediction models may support early identification and targeted prevention, but their methodological quality, validation rigor, and clinical readiness remain uncertain.

**Objective:**

This systematic review aimed to synthesize and critically evaluate prediction models for in-hospital delirium developed using routinely collected EHR data, focusing on model characteristics, validation strategies, performance, risk of bias, and clinical applicability.

**Methods:**

We searched PubMed, MEDLINE, Embase, PsycINFO, and Web of Science from inception to November 11, 2025. Eligible studies developed, validated, or evaluated multivariable prediction models using routinely collected EHR or administrative data to predict acute mental status deterioration during adult hospital admissions. Although eligibility criteria were broad, all included studies operationalized deterioration as delirium. Data extraction was informed by CHARMS (Checklist for Critical Appraisal and Data Extraction for Systematic Reviews of Prediction Modeling Studies) and TRIPOD (Transparent Reporting of a Multivariable Prediction Model for Individual Prognosis or Diagnosis) or TRIPOD–artificial intelligence guidance. Model performance, validation, calibration, and implementation features were synthesized narratively. Risk of bias and applicability were assessed using PROBAST (Prediction Model Risk of Bias Assessment Tool).

**Results:**

Twenty-nine studies met the inclusion criteria. The evidence clustered into 4 overlapping prediction tasks: admission or early-stay risk stratification, perioperative or postoperative prediction, dynamic intensive care unit prediction, and external validation or workflow evaluation of existing tools. Most studies were retrospective cohorts (20/29, 69%) and were conducted in general ward, mixed ward–intensive care unit, intensive care unit, or emergency department settings. Machine learning or hybrid approaches were common (18/29, 62%), but more complex models did not consistently outperform statistical or rule-based approaches. Of 29 studies, internal discrimination was reported in 24 (83%; area under the receiver operating characteristic curve range 0.77-0.97) studies, whereas external discrimination was reported in 12 studies and calibration in 15 studies. Decision curve analysis was reported in 3 studies, and prospective evaluation or workflow integration remained limited. Overall risk of bias was low in 8 studies, unclear in 10 studies, and high in 11 studies, mainly because of analysis-domain limitations.

**Conclusions:**

Routinely collected EHR data can support delirium risk prediction across hospital settings, and many models show moderate to high discrimination. However, no single algorithm is ready for routine adoption. The field remains limited by heterogeneous prediction tasks, inconsistent outcome ascertainment, weak calibration and decision-analytic reporting, and insufficient external or prospective evaluation. Future studies should define the intended clinical use case before model development, evaluate calibration and clinical usefulness alongside discrimination, and test models across institutions, time periods, and workflows before deployment.

## Introduction

Acute in-hospital mental status deterioration is a clinically important manifestation of acute brain dysfunction, encompassing disturbances such as confusion, inattention, agitation, and reduced consciousness. In current hospital-based prediction modeling research using routinely collected electronic health record (EHR) data, however, this construct has been operationalized almost exclusively as delirium. Delirium is common across hospital settings and is associated with increased morbidity, mortality, prolonged hospitalization, institutionalization, and persistent cognitive impairment following discharge [1,2].

Delirium is the most clinically and methodologically established target in this literature. Its prominence reflects both its prognostic significance and the availability of validated bedside assessment tools, most notably the Confusion Assessment Method (CAM) and its intensive care unit (ICU) adaptations [3].

Prediction modeling for in-hospital delirium is motivated by the need to support early identification and prevention in resource-constrained clinical environments. Universal application of intensive preventive strategies is rarely feasible, and risk stratification tools that identify patients at elevated risk early in the hospital course offer a pragmatic approach to targeting preventive interventions [4]. Advances in EHR-based modeling, including machine learning and natural language processing (NLP), have enabled scalable development of delirium prediction models using routinely collected clinical data [5].

However, recent systematic reviews have highlighted important limitations in the existing evidence base. Although many delirium prediction models report moderate to high discrimination, substantial heterogeneity exists in outcome definitions, predictor handling, validation strategies, and reporting quality. External validation, calibration assessment, and prospective evaluation remain inconsistently performed, limiting confidence in clinical generalizability and real-world usefulness [6-8].

Against this background, the present systematic review aims to synthesize and critically evaluate prediction models for in-hospital delirium using routinely collected EHR data. Although the search strategy was intentionally broad to capture a range of acute mental status outcomes, all eligible studies identified at full-text review operationalized deterioration as delirium. This finding underscores the central role of delirium as the dominant and currently most clinically actionable manifestation of acute in-hospital mental status change within the prediction modeling literature. Accordingly, this review focuses on delirium prediction models, with particular emphasis on methodological quality, validation practices, risk of bias, and implications for the development of clinically actionable decision support tools.

## Methods

### Study Design and Reporting Standards

This systematic review was conducted and reported in accordance with the PRISMA (Preferred Reporting Items for Systematic Reviews and Meta-Analyses) guidelines [9]. Given the focus on prediction model development and validation, data extraction and synthesis were additionally informed by relevant items from the TRIPOD (Transparent Reporting of a Multivariable Prediction Model for Individual Prognosis or Diagnosis) and TRIPOD–Artificial Intelligence (TRIPOD-AI) reporting frameworks to support structured evaluation of model characteristics, validation strategies, and reporting quality [10,11]. This review was not prospectively registered in PROSPERO because protocol registration was not completed before screening had begun; this is acknowledged as a limitation.

### Eligibility Criteria

Studies were eligible for inclusion if they met the following criteria: (1) involved adult patients (aged ≥18 years) admitted to hospital settings, including ICUs, general medical or surgical wards, or mixed ICU–ward cohorts; (2) developed, validated, or updated multivariable prediction models using routinely collected EHR or administrative data; (3) predicted delirium occurring during the index hospital admission; and (4) reported sufficient methodological or performance information to allow data extraction.

Eligible outcomes included delirium occurring during the index hospital admission, identified using validated clinical assessment tools (eg, CAM and CAM-ICU), diagnostic codes, or structured chart review. Although the search strategy was designed to capture a broader range of acute mental status deterioration outcomes, all studies meeting inclusion criteria at full-text review predicted delirium. Therefore, delirium was treated as the primary outcome for this review.

Studies using any statistical or machine learning–based modeling approach were eligible, including traditional regression-based models and advanced machine learning methods. Both retrospective and prospective study designs were included. Studies were excluded if they (1) focused exclusively on pediatric populations; (2) were conducted in outpatient, community, or psychiatric clinic settings without hospital admission; (3) relied solely on nonroutine data sources such as imaging, genomics, or specialized psychometric instruments not typically available in EHR systems; (4) predicted outcomes defined at the population level or occurring outside the index hospital admission (eg, long-term suicide risk or postdischarge psychiatric readmission); or (5) did not report delirium or an equivalent acute in-hospital mental status outcome.

### Information Sources and Search Strategy

A comprehensive literature search was conducted to identify studies developing or validating prediction models for delirium and related acute mental status outcomes occurring during hospitalization. The search strategy was designed a priori to be intentionally broad to maximize sensitivity and capture models targeting a range of clinically relevant mental status changes. However, all studies meeting inclusion criteria at full-text review focused exclusively on delirium. This reflects the predominance of delirium as the most consistently defined and operationalized outcome in this field, and the present review therefore focuses specifically on delirium prediction models.

The following electronic databases were searched from inception to November 11, 2025: PubMed or MEDLINE, Embase, PsycINFO, and Web of Science. The search combined terms related to prediction modeling and machine learning (eg, prediction, risk model, machine learning, and AI), routinely collected health care data (eg, EHRs, administrative data, and claims data), acute mental status outcomes (eg, delirium, acute confusion, agitation, mental status change, and psychotropic medication use), and hospital settings (eg, inpatient, ward, and ICU).

Database-specific search strategies were developed using a combination of controlled vocabulary (eg, MeSH) and free-text terms. Full search strategies for each database are provided in Multimedia Appendix 1. No restrictions were placed on geographic location or health care setting. Only studies published in English were included. Reference lists of included studies and relevant review papers were manually screened to identify additional eligible studies. All retrieved records were imported into reference management software, and duplicate records were removed before screening.

### Study Selection

After removal of duplicate records, titles and abstracts were screened to identify potentially eligible studies. Full-text papers were retrieved for all records deemed potentially relevant and assessed against the predefined eligibility criteria. Study selection was performed independently by 2 reviewers (CSL and GWC). Discrepancies at either the title or abstract or full-text screening stage were resolved through discussion and consensus, with consultation of a third reviewer (MHT) when necessary. The overall study selection process is summarized using a PRISMA flow diagram.

### Data Extraction

Data were extracted independently by 2 reviewers (CSL and GWC) using a standardized data extraction form developed a priori. The extraction framework was informed by established guidance for prediction model studies, including the CHARMS (Checklist for Critical Appraisal and Data Extraction for Systematic Reviews of Prediction Modelling Studies), and reporting domains from the TRIPOD and TRIPOD-AI statements [10-12].

For each included study, extracted information included study metadata (first author, year, country, clinical setting, and study design), population characteristics (sample size and cohort definition), outcome definition and ascertainment method (eg, CAM-based assessment, *ICD* [*International Classification of Diseases*] codes, and chart review), prediction time horizon, modeling approach, predictor variables, feature selection methods, and model presentation.

Model development and validation characteristics were extracted in detail, including type of validation (internal, temporal, or external), validation datasets, and reported performance measures. Extracted performance metrics included measures of discrimination (eg, area under the receiver operating characteristic curve [AUROC]), sensitivity, specificity, and predictive values where available. Calibration measures and decision-analytic measures (eg, decision curve analysis) were recorded when reported. Information on missing data handling was extracted for each study, including use of complete-case analysis, single or multiple imputation, or algorithm-intrinsic handling of missingness. Where missing data handling was not explicitly described, this was recorded as not reported.

### Risk of Bias and Quality Assessment

Risk of bias and applicability of included studies were assessed using the PROBAST (Prediction Model Risk of Bias Assessment Tool), which evaluates 4 domains: participants, predictors, outcomes, and analysis [13]. Two reviewers (CSL and GWC) independently completed PROBAST assessments using the tool’s signaling questions. Each domain was rated as low, high, or unclear risk of bias. Discrepancies were resolved through discussion and consensus, with involvement of a third reviewer (MHT) when necessary. PROBAST assessments were used to support qualitative interpretation of methodological strengths and limitations rather than to exclude studies or weight quantitative comparisons.

### Data Synthesis and Analysis

Given substantial heterogeneity across studies with respect to clinical setting, population characteristics, outcome definitions, prediction horizons, modeling approaches, and validation strategies, a quantitative meta-analysis of model performance was not performed. Findings were therefore synthesized narratively using a structured framework focused on 4 questions: What clinical prediction task was being addressed? What routinely collected data were used? How well models performed under internal and external validation? How close the models were to clinically reliable implementation.

Prediction model characteristics and performance metrics were summarized descriptively. Performance differences were interpreted in relation to clinical setting, prediction horizon, outcome ascertainment, validation strategy, calibration reporting, and risk of bias rather than by algorithm type alone. This approach was chosen to distinguish technical feasibility from clinical readiness.

## Results

### Study Selection

**Figure 1.**
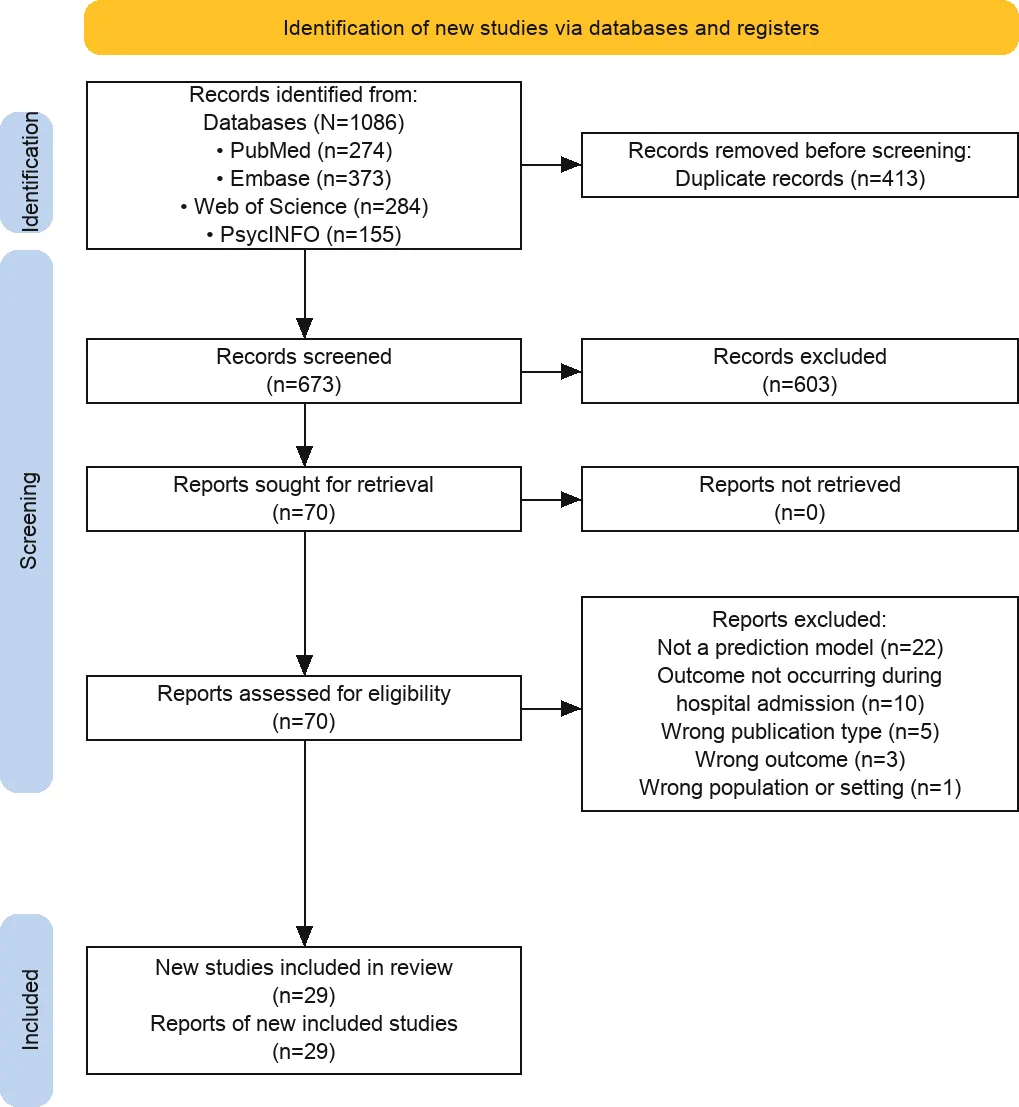
PRISMA (Preferred Reporting Items for Systematic Reviews and Meta-Analyses) flow diagram of study selection [9].

The database search identified a total of 1086 records, including 274 from PubMed, 373 from Embase, 284 from Web of Science, and 155 from PsycINFO. After removal of duplicate and overlapping records, 673 unique records remained for title and abstract screening, of which 603 were excluded. Seventy reports were retrieved for full-text assessment. Following full-text review, 41 reports were excluded. The most common reasons for exclusion were the absence of a multivariable prediction model, outcomes not occurring during the index hospital admission, wrong publication type, wrong outcome definition, or an ineligible study population. A total of 29 studies met the inclusion criteria and were included in the final qualitative synthesis. The study selection process is summarized in Figure 1.

### Study Characteristics

The 29 included studies did not represent a single uniform prediction problem. Instead, they formed a heterogeneous evidence base spanning early admission screening, perioperative risk prediction, short-term ICU warning systems, and external validation or prospective evaluation of existing tools. Country, center structure, and study design are summarized in Table 1. The narrative synthesis highlights the patterns most relevant to interpretation.

**Table 1.** Study characteristics (country, center, and study design).

Study ID	Country	Centers	Study design
Ali et al (2023) [14]	Netherlands	Single-center	Retrospective cohort
Bartolacci et al (2025) [15]	United States	Single-center	Retrospective cohort
Bishara et al (2022) [16]	United States	Multicenter	Retrospective cohort
Castro et al (2021) [17]	United States	Mixed (multicenter+external validation)	Retrospective cohort
Ceppi et al (2023) [18]	Switzerland	Single-center	Retrospective cohort
Contreras et al (2025) [19]	United States	Multicenter	Retrospective cohort
Contreras et al (2023) [20]	United States	Single-center	Retrospective cohort
Corradi et al (2018) [21]	United States	Single-center	Retrospective cohort
Davoudi et al (2017) [22]	United States	Single-center	Retrospective cohort
Heikal et al (2024) [23]	Lebanon/United States	Mixed (multicenter+external validation)	Retrospective cohort
Holler et al (2025) [24]	United States	Multicenter	Retrospective case–control
Hur et al (2021) [25]	South Korea	Mixed (multicenter+external validation)	Retrospective cohort
Jauk et al (2020) [26]	Austria	Single-center	Prospective cohort
Jauk et al (2022) [27]	Austria	Mixed (multicenter+external validation)	Mixed retrospective–prospective
Jauk et al (2024) [28]	Austria	Single-center	Prospective cohort
Jung et al (2022) [29]	South Korea	Multicenter	Retrospective cohort
Li et al (2024) [30]	China	Single-center	Retrospective cohort
Liu et al (2022) [31]	United States	Single-center	Retrospective cohort
Lucini et al (2023) [32]	Canada	Multicenter	Retrospective cohort
Matsumoto et al (2023) [33]	Japan	Single-center	Retrospective cohort
Moon et al (2018) [34]	South Korea	Mixed (multicenter+external validation)	Mixed retrospective–prospective
Mueller et al (2023) [35]	United States	Single-center	Retrospective cohort
Pagali et al (2022) [36]	United States	Single-center	Prospective cohort
Reeve et al (2025) [37]	Switzerland	Single-center	Prospective cohort
Rudolph et al (2016) [38]	United States	Mixed (multicenter+external validation)	Mixed retrospective–prospective
Sheikhalishahi et al (2023) [39]	United States	Mixed (multicenter+external validation)	Retrospective cohort
Sun et al (2021) [40]	Germany	Multicenter	Retrospective cohort
Sun et al (2022) [41]	Germany	Multicenter	Mixed retrospective–prospective
Wong et al (2018) [42]	United States	Single-center	Retrospective cohort

Most studies used retrospective cohort designs (20/29, 69%) [14-23,25,29-33,35,39,40,42]. Of 29 studies, prospective cohorts were less common (4/29, 14%) [26,28,36,37], 4 (14%) studies combined retrospective development with prospective validation or evaluation [27,34,38,41], and 1 (3%) study used a retrospective case-control design [24].

This design profile is important because much of the literature remains closer to model development than to implementation science. The strongest clinical evidence generally came from studies that moved beyond internal validation into external, temporal, or prospective evaluation.

Clinical setting, population focus, and sample size are summarized in Table 2. Clinical settings were unevenly represented. Of 29 studies, general ward populations accounted for 13 (45%) studies, mixed ward-ICU cohorts accounted for 8 (28%), ICU-only cohorts accounted for 7 (24%), and the emergency department accounted for 1 (3%). Fifteen (52%) studies were single-center, while 14 (48%) studies used multicenter data or combined multicenter development with external validation.

**Table 2.** Clinical setting, population focus, and sample size.

Study ID	Clinical setting	Population focus	Total, N
Ali et al (2023) [14]	General ward	Older adult inpatients (≥60 years)	1168 patients
Bartolacci et al (2025) [15]	Emergency department	Older adult inpatients (≥65 years)	44,578 patients
Bishara et al (2022) [16]	Mixed ICU^a^ and ward	Adult surgical inpatients	24,885 encounters
Castro et al (2021) [17]	Mixed ICU and ward	Adult medical inpatients (COVID-19)	2907 patients
Ceppi et al (2023) [18]	General ward	Adult rehabilitation inpatients	8774 stays
Contreras et al (2025) [19]	ICU	Adult ICU patients	104,303 patients
Contreras et al (2023) [20]	ICU	Adult ICU patients	13,395 patients
Corradi et al (2018) [21]	Mixed ICU and ward	Adult general inpatients	41,826 patients
Davoudi et al (2017) [22]	General ward	Adult surgical inpatients	51,457 patients
Heikal et al (2024) [23]	Mixed ICU and ward	Adult inpatients (ICU and ward)	40,208 admissions
Holler et al (2025) [24]	General ward	Older adult surgical inpatients (≥50 years)	14,334 encounters
Hur et al (2021) [25]	ICU	Adult ICU patients	12,409 patients
Jauk et al (2020) [26]	General ward	Adult general inpatients	4663 patients
Jauk et al (2022) [27]	General ward	Adult trauma surgery inpatients	93 patients
Jauk et al (2024) [28]	General ward	Adult surgical inpatients	738 patients
Jung et al (2022) [29]	General ward	Older adult orthopedic surgery inpatients	3980 patients
Li et al (2024) [30]	ICU	Adult cardiac surgery inpatients	507 patients
Liu et al (2022) [31]	Mixed ICU and ward	Adult general inpatients	34,035 patients
Lucini et al (2023) [32]	ICU	Adult ICU patients	38,426 patients
Matsumoto et al (2023) [33]	General ward	Adult surgical inpatients	11,863 patients
Moon et al (2018) [34]	ICU	Adult ICU patients	3284 patients
Mueller et al (2023) [35]	Mixed ICU and ward	Older adult inpatients (ED^b^ admission)	28,531 patients
Pagali et al (2022) [36]	Mixed ICU and ward	Older adult inpatients (≥50 years)	8055 patients
Reeve et al (2025) [37]	General ward	Older adult surgical inpatients (≥60 years)	866 patients
Rudolph et al (2016) [38]	General ward	Older adult inpatients	27,871 patients
Sheikhalishahi et al (2023) [39]	ICU	Adult ICU patients	22,840 patients
Sun et al (2021) [40]	General ward	Adult general inpatients	NR^c^
Sun et al (2022) [41]	Mixed ICU and ward	Adult general inpatients	NR
Wong et al (2018) [42]	General ward	Adult general inpatients	18,223

a ICU: intensive care unit.

b ED: emergency department.

c NR: not reported.

Across all studies, adult inpatients constituted the primary population of interest, but the intended use cases differed. Some models were designed for broad hospital screening, some for older adult or surgical pathways, and others for high-frequency monitoring in intensive care. Sample sizes ranged from fewer than 100 patients in a small prospective deployment cohort [27] to more than 100,000 patients in large multidatabase studies [19], making direct comparison of performance estimates difficult.

Outcome definitions, assessment methods, and prevalence are summarized in Table 3. Delirium was the outcome across all included studies, but the clinical meaning of the outcome varied. Of the 29 studies, 27 (93%) modeled incident delirium, 1 (3%) modeled prevalent delirium in the emergency department [15], and 1 (3%) considered any delirium regardless of timing [38]. Outcome labels included in-hospital delirium, postoperative delirium, ICU delirium, and recurrent or short-term delirium risk.

**Table 3.** Outcome definition, assessment method, and prevalence.

Study ID	Outcome label	Assessment method	Outcome prevalence
Ali et al (2023) [14]	In-hospital delirium	Chart review or adjudication	75/1345 (5.6%)
Bartolacci et al (2025) [15]	In-hospital delirium	CAM^a^-based screening	1701/44,578 (3.8%)
Bishara et al (2022) [16]	Postoperative delirium	Nurse routine screening (CAM-based)	1327/24,885 (5.3%)
Castro et al (2021) [17]	Incident delirium	EHR^b^-derived (codes or NLP^c^)	488/2907 (16.8%)
Ceppi et al (2023) [18]	Incident delirium	EHR-derived+chart review	125/8774 (1.4%)
Contreras et al (2025) [19]	Incident delirium	CAM-ICU^d^	Reported without extractable event count
Contreras et al (2023) [20]	ICU delirium	CAM-ICU	12,871/56,297 windows (23%)
Corradi et al (2018) [21]	Incident delirium	Nurse routine screening (CAM-based)	3499/64,038 visits (5.5%)
Davoudi et al (2017) [22]	Postoperative delirium	ICD^e^ codes	1608/51,457 (3.1%)
Heikal et al (2024) [23]	ICU delirium or In-hospital delirium	*ICD* codes+chart review	NR^f^
Holler et al (2025) [24]	Postoperative delirium	CAM-based+*ICD* codes	7198/39,968 raw cohort (18.0%)
Hur et al (2021) [25]	ICU delirium	CAM-ICU	Reported by dataset; event counts NR
Jauk et al (2020) [26]	In-hospital delirium	*ICD* codes+EHR text review	81/5530 (1.5%)
Jauk et al (2022) [27]	In-hospital delirium	Clinical judgment/chart review	NR clinical cohort; 347/5347 external test set
Jauk et al (2024) [28]	In-hospital delirium	DOS^g^	103/738 (14%)
Jung et al (2022) [29]	Postoperative delirium	DSM^h^-based diagnosis+EHR-derived	196/3980 (4.9%)
Li et al (2024) [30]	Postoperative delirium	CAM-ICU	141/507 (28%)
Liu et al (2022) [31]	Incident delirium	CAM-ICU	Positive CAM assessment rate reported; event count NR
Lucini et al (2023) [32]	ICU delirium	ICDSC^i^	Episode prevalence reported; event count NR
Matsumoto et al (2023) [33]	Postoperative delirium	CAM-based screening	592/6497 derivation (9.1%); 427/5366 validation (8.0%)
Moon et al (2018) [34]	ICU delirium	CAM-ICU	688/3284 (21%)
Mueller et al (2023) [35]	In-hospital delirium	DOS/CAM-ICU	8057/28,351 (28.4%)
Pagali et al (2022) [36]	In-hospital delirium	CAM-based screening	1107/8055 (13.7%)
Reeve et al (2025) [37]	Postoperative delirium	DOS+*ICD* codes	100/866 (11.5%)
Rudolph et al (2016) [38]	In-hospital delirium	DSM-based diagnosis+EHR-derived	2343/27,625 retrospective (8%); 43/246 prospective incident (19%)
Sheikhalishahi et al (2023) [39]	Incident delirium	CAM-ICU	NR
Sun et al (2021) [40]	In-hospital delirium	*ICD* codes	NR
Sun et al (2022) [41]	In-hospital delirium	*ICD* codes	NR
Wong et al (2018) [42]	Incident delirium	Nurse routine screening (CAM-based)	878/18,223 (4.8%)

a CAM: Confusion Assessment Method.

b EHR: electronic health record.

c NLP: natural language processing.

d ICU: intensive care unit.

e *ICD*: *International Classification of Diseases*.

f NR: not reported.

g DOS: Delirium Observation Screening Scale.

h DSM: Diagnostic and Statistical Manual of Mental Disorders.

i ICDSC: Intensive Care Delirium Screening Checklist.

Outcome ascertainment was a major source of heterogeneity. Studies used structured tools such as CAM, CAM-ICU, the Delirium Observation Screening Scale, or the Intensive Care Delirium Screening Checklist, as well as *ICD* codes, EHR-derived definitions, NLP-enhanced ascertainment, and manual chart review or adjudication. These approaches identify overlapping but not identical clinical events, which limits the interpretability of pooled performance comparisons.

Outcome prevalence also varied substantially. Among the 20 studies with clearly extractable prevalence percentages, 6 (30%) reported prevalence below 5%, 10 (50%) reported prevalence between 5% and 20%, and 4 (20%) reported prevalence above 20%. Low prevalence was typical in general ward and broad inpatient cohorts, whereas higher prevalence was more common in ICU, surgical, and selected high-risk cohorts.

This variation has direct implications for clinical interpretation. In low-prevalence settings, even a model with good AUROC may have low positive predictive value (PPV) and may generate many false-positive alerts. In higher-prevalence settings, the same threshold can produce a very different balance between missed cases and unnecessary intervention.

Prediction horizon and TRIPOD classification are summarized in Table 4. Prediction horizons further separated the studies into different clinical tasks. Some models estimated risk at admission or during the first 24‐72 hours, some predicted postoperative delirium over a fixed perioperative period, and others updated risk dynamically using rolling ICU or hospital windows. These are not interchangeable tasks: short-term dynamic prediction benefits from more proximal clinical signals, whereas admission-time models must support earlier but less certain prevention decisions.

**Table 4.** Prediction horizon and TRIPOD^a^ classification.

Study ID	Prediction horizon	TRIPOD type
Ali et al (2023) [14]	During hospitalization	TRIPOD 4
Bartolacci et al (2025) [15]	During ED^b^ stay	TRIPOD 4
Bishara et al (2022) [16]	Postoperative period (≤7 days)	TRIPOD 1b
Castro et al (2021) [17]	During hospitalization	TRIPOD 2b
Ceppi et al (2023) [18]	During hospitalization	TRIPOD 1a
Contreras et al (2025) [19]	During ICU^c^ stay	TRIPOD 2b
Contreras et al (2023) [20]	Dynamic short-term window (≤24 hours)	TRIPOD 1b
Corradi et al (2018) [21]	During hospitalization	TRIPOD 1b
Davoudi et al (2017) [22]	Postoperative period (≤7 days)	TRIPOD 1b
Heikal et al (2024) [23]	During ICU stay/During hospitalization	TRIPOD 2a
Holler et al (2025) [24]	Postoperative period (≤7 days)	TRIPOD 2b
Hur et al (2021) [25]	Early admission window (≤24‐72 hours)	TRIPOD 2b
Jauk et al (2020) [26]	During hospitalization	TRIPOD 1b
Jauk et al (2022) [27]	Early admission window (≤24‐72 hours)	TRIPOD 3
Jauk et al (2024) [28]	Postoperative period (≤7 days)	TRIPOD 1b
Jung et al (2022) [29]	Postoperative period (≤7 days)	TRIPOD 2b
Li et al (2024) [30]	Postoperative period (≤7 days)	TRIPOD 1b
Liu et al (2022) [31]	Dynamic short-term window (≤24 hours)	TRIPOD 1b
Lucini et al (2023) [32]	Dynamic short-term window (≤24 hours)	TRIPOD 1b
Matsumoto et al (2023) [33]	During hospitalization	TRIPOD 2b
Moon et al (2018) [34]	During ICU stay	TRIPOD 2b
Mueller et al (2023) [35]	Early admission window (≤24‐72 hours)	TRIPOD 1b
Pagali et al (2022) [36]	During hospitalization	TRIPOD 4
Reeve et al (2025) [37]	Postoperative period (≤7 days)	TRIPOD 4
Rudolph et al (2016) [38]	During hospitalization	TRIPOD 2b
Sheikhalishahi et al (2023) [39]	Dynamic short-term window (≤48 hours)	TRIPOD 1b
Sun et al (2021) [40]	During hospitalization	TRIPOD 2b
Sun et al (2022) [41]	During hospitalization	TRIPOD 2b
Wong et al (2018) [42]	During hospitalization	TRIPOD 2a

a TRIPOD: Transparent Reporting of a Multivariable Prediction Model for Individual Prognosis or Diagnosis.

b ED: emergency department.

c ICU: intensive care unit.

Accordingly, apparent differences in discrimination should not be interpreted as simple evidence that one model family is superior. They may instead reflect differences in timing, population acuity, outcome prevalence, and availability of predictors close to delirium onset.

TRIPOD classifications also reflected a field still weighted toward development and validation rather than deployment. Type 1b and 2b studies predominated, while fewer studies focused on external validation, model updating, temporal validation, or prospective implementation.

Taken together, the study characteristics show that the literature is clinically broad but methodologically fragmented. The central synthesis question is therefore not only whether EHR-based delirium prediction is feasible but which models have been tested under conditions close enough to their intended clinical use.

### Model Development and Predictors

Model categories are summarized in Table 5, with detailed model development and predictor characteristics provided in Multimedia Appendix 2 [14-42]. Machine learning models were the largest single group (10/29, 34%), followed by statistical or rule-based approaches (8/29, 28%), deep learning models (5/29, 17%), and hybrid approaches combining statistical, machine learning, or deep learning components (6/29, 21%). Tree-based ensemble methods such as random forests and gradient boosting were common, while deep learning was concentrated in ICU or large-scale EHR datasets.

**Table 5.** Model category across included studies.

Study ID	Model category
Ali et al (2023) [14]	Existing rule-based statistical model (external validation only)
Bartolacci et al (2025) [15]	Not applicable (model validation study; no development)
Bishara et al (2022) [16]	Classical machine learning and neural network models with clinical score comparator
Castro et al (2021) [17]	Penalized logistic regression with simple clinical comparators
Ceppi et al (2023) [18]	Multivariable logistic regression
Contreras et al (2025)[19]	Deep learning models including transformers and large language models
Contreras et al (2023) [20]	Tree-based machine learning and recurrent neural networks
Corradi et al (2018) [21]	Tree-based machine learning (Random Forest)
Davoudi et al (2017) [22]	Classical machine learning and statistical models
Heikal et al (2024) [23]	Classical and ensemble machine learning models
Holler et al (2025) [24]	Classical machine learning and neural networks
Hur et al (2021) [25]	Classical machine learning and deep neural networks
Jauk et al (2020) [26]	Tree-based machine learning with multiple outcome models
Jauk et al (2022) [27]	Tree-based machine learning
Jauk et al (2024) [28]	Tree-based machine learning
Jung et al (2022) [29]	Gradient boosting models
Li et al (2024) [30]	Classical machine learning models
Liu et al (2022) [31]	Hybrid machine learning and deep learning models
Lucini et al (2023) [32]	Recurrent neural networks
Matsumoto et al (2023) [33]	Gradient boosting and penalized regression
Moon et al (2018) [34]	Multivariable logistic regression
Mueller et al (2023) [35]	Classical machine learning models
Pagali et al (2022) [36]	Rule-based model with statistical recalibration
Reeve et al (2025) [37]	External validation of existing model
Rudolph et al (2016) [38]	Rule-based statistical model
Sheikhalishahi et al (2023) [39]	Deep learning with attention mechanisms
Sun et al (2021) [40]	Transformer-based deep learning
Sun et al (2022) [41]	Transformer-based deep learning
Wong et al (2018) [42]	Classical machine learning and statistical models

Several studies compared multiple modeling paradigms within the same dataset [16,17,20,22,24,25,31,33,42]. These comparisons did not show a consistent advantage for increasing model complexity. In some cohorts, tree-based or penalized regression models performed similarly to neural network approaches, and apparent internal advantages were not always preserved during external validation.

Predictor sets were built mainly from structured EHR data. Of 29 studies, demographics were included in 28 (97%), comorbidities or diagnostic history in 25 (86%) , laboratory values in 22 (76%), medications in 20 (69%), vital signs in 17 (59%), and nursing or care process variables in 7 (24%). This pattern indicates that most models relied on broadly available routine data rather than specialized research measurements.

Temporal handling of predictors was a key distinction between models (Table 6). Of 29 studies, 15 (52%) used static single-time snapshots, most often at admission, preoperatively, or within an early fixed window. Of 29 studies, 14 (48%) incorporated time-varying information through repeated recalculation, aggregated temporal summaries, rolling windows, or sequential time series modeling.

**Table 6.** Predictor window and temporal handling.

Study ID	Predictor window	Temporal handling
Ali et al (2023) [14]	Admission-time or early admission window (≤24 hours)	Static with repeated recalculation
Bartolacci et al (2025) [15]	ED^a^ presentation window	Static (single-time snapshot)
Bishara et al (2022) [16]	Preoperative window	Static (single-time snapshot)
Castro et al (2021) [17]	Preadmission+ early admission window	Static (single-time snapshot)
Ceppi et al (2023) [18]	Admission-time window	Static (single-time snapshot)
Contreras et al (2025) [19]	Early ICU^b^ window (≤24 hours)	Aggregated temporal summaries
Contreras et al (2023) [20]	Mixed static+short-term dynamic ICU window	Hybrid static+temporal modeling
Corradi et al (2018) [21]	Dynamic ICU window (until outcome)	Aggregated temporal summaries
Davoudi et al (2017) [22]	Preoperative window	Static (single-time snapshot)
Heikal et al (2024) [23]	Dynamic ICU window or dynamic hospital window	Aggregated temporal summaries
Holler et al (2025) [24]	Preadmission window	Static (single-time snapshot)
Hur et al (2021) [25]	Early ICU window (≤24 hours)	Aggregated temporal summaries
Jauk et al (2020) [26]	Admission-time+early admission window	Static with repeated recalculation
Jauk et al (2022) [27]	Preadmission+early admission window	Static (single-time snapshot)
Jauk et al (2024) [28]	Early admission window (≤48 hours)	Static with repeated recalculation
Jung et al (2022) [29]	Preoperative window	Static (single-time snapshot)
Li et al (2024) [30]	Preoperative+perioperative+early ICU window	Static (single-time snapshot)
Liu et al (2022) [31]	Short-term dynamic window (≤24 hours)	Hybrid static+temporal modeling
Lucini et al (2023) [32]	Mixed static+dynamic ICU window	Sliding or rolling time windows
Matsumoto et al (2023) [33]	Admission-time+perioperative window	Static (single-time snapshot)
Moon et al (2018) [34]	Early ICU window (≤24 hours)	Static (single-time snapshot)
Mueller et al (2023) [35]	ED presentation+early admission window	Static (single-time snapshot)
Pagali et al (2022) [36]	Admission-time or early admission window	Static (single-time snapshot)
Reeve et al (2025) [37]	Preoperative window	Static (single-time snapshot)
Rudolph et al (2016) [38]	Early admission window (≤24 hours)	Aggregated temporal summaries
Sheikhalishahi et al (2023) [39]	Short-term dynamic window (≤24 hours)	Sequential time series modeling
Sun et al (2021) [40]	Admission-time+dynamic hospital window	Aggregated temporal summaries
Sun et al (2022) [41]	Dynamic hospital window (entire stay)	Aggregated temporal summaries
Wong et al (2018) [42]	Early admission window (≤24 hours)	Static (single-time snapshot)

a ED: emergency department.

b ICU: intensive care unit.

Of 29 studies, only 8 (28%) used NLP or unstructured free-text data [17,18,23,26,29,38,40,41]. In several of these, NLP supported outcome ascertainment rather than contributing predictor features. Thus, despite the clinical importance of narrative documentation for cognitive and behavioral change, most models remained dependent on structured EHR fields.

Reporting of missing data handling, class imbalance, and interpretability was uneven (Table 7). Missing data handling was not reported in 24% (7/29) of the studies, and class imbalance handling was absent or not reported in 55% (16/29) of the studies. Interpretability was addressed most often through post hoc feature attribution (14/29, 48%) or intrinsically interpretable models (7/29, 24%), but these explanations were rarely linked to clinical workflow decisions.

**Table 7.** Missing data handling and model interpretability.

Study ID	Missing data handling	Interpretability approach
Ali et al (2023) [14]	No imputation or model design–based	None or not reported
Bartolacci et al (2025) [15]	Simple imputation	Intrinsic interpretability
Bishara et al (2022) [16]	Simple imputation	Hybrid (intrinsic+post hoc)
Castro et al (2021) [17]	Simple imputation+complete-case	Intrinsic interpretability
Ceppi et al (2023) [18]	Exclusion-based (no imputation)	Intrinsic interpretability
Contreras et al (2025) [19]	Simple imputation+missingness indicators	Post hoc feature attribution
Contreras et al (2023) [20]	Time series imputation	Post hoc feature attribution
Corradi et al (2018) [21]	Model-intrinsic handling	Post hoc feature attribution
Davoudi et al (2017) [22]	Imputation (method not specified)	Post hoc feature attribution
Heikal et al (2024) [23]	Advanced imputation (DL^a^-based)	Post hoc feature attribution
Holler et al (2025) [24]	Exclusion-based (no imputation)	Post hoc feature attribution
Hur et al (2021) [25]	Simple imputation	None or not reported
Jauk et al (2020) [26]	Not reported	Limited or unclear
Jauk et al (2022) [27]	Not reported	Post hoc feature attribution
Jauk et al (2024) [28]	Not reported	Post hoc feature attribution
Jung et al (2022) [29]	Model-intrinsic handling	Post hoc feature attribution
Li et al (2024) [30]	Simple imputation+exclusions	Post hoc feature attribution
Liu et al (2022) [31]	Simple imputation	Post hoc feature attribution
Lucini et al (2023) [32]	Simple imputation + missingness indicators	Post hoc feature attribution
Matsumoto et al (2023) [33]	Advanced imputation (ML^b^-based)	Hybrid (intrinsic+post hoc)
Moon et al (2018) [34]	Not reported	Intrinsic interpretability
Mueller et al (2023) [35]	Advanced imputation (ML-based)	Hybrid (intrinsic+post hoc)
Pagali et al (2022) [36]	Simple imputation	Intrinsic interpretability
Reeve et al (2025) [37]	Simple imputation	Intrinsic interpretability
Rudolph et al (2016) [38]	Not reported	Intrinsic interpretability
Sheikhalishahi et al (2023) [39]	Time series imputation	Attention-based or ante hoc
Sun et al (2021) [40]	Not reported	Post hoc feature attribution
Sun et al (2022) [41]	Not reported	Limited or unclear
Wong et al (2018) [42]	Simple imputation+missingness indicators	Post hoc feature attribution

a DL: deep learning.

b ML: machine learning.

Overall, model development methods show that EHR-based delirium prediction is technically feasible, but the clinical value of additional data complexity remains uncertain without stronger validation, calibration, and implementation testing.

### Model Performance

Model discrimination is summarized in Table 8, with additional model performance metrics, including sensitivity, specificity, PPV, negative predictive value (NPV), and threshold definitions provided in Multimedia Appendix 3 [14-42]. Reported discrimination suggested promising technical performance but should be interpreted cautiously. Of 29 studies, internal AUROC was reported in 24 (83%) studies, with values ranging from 0.77 [24] to 0.97 [23]. Confidence intervals were inconsistently reported, and the internal estimates came from heterogeneous designs, including split-sample validation, cross-validation, temporal evaluation, and prospective cohorts.

**Table 8.** Model discrimination (internal and external AUROC^a^).

Study ID	Internal AUROC	External AUROC
Ali et al (2023) [14]	Not reported	Not reported
Bartolacci et al (2025) [15]	Not applicable	Kennedy: 0.777; Zucchelli: 0.701; MDP^b^: 0.898; REDEEM^c^: 0.921
Bishara et al (2022) [16]	XGBoost:^d^ 0.851; Neural network: 0.841	Not performed
Castro et al (2021) [17]	Not reported	0.75 (95% CI 0.71‐0.79)
Ceppi et al (2023) [18]	0.917	Not performed
Contreras et al (2025) [19]	0.848 (95% CI 0.818‐0.878)	0.824 (95% CI 0.818‐0.830)
Contreras et al (2023) [20]	0.87 (95% CI 0.86‐0.87; CatBoost on evaluation set)	Not performed
Corradi et al (2018) [21]	0.909 (95% CI 0.898‐0.921)	Not performed
Davoudi et al (2017) [22]	−0.85 to 0.86 (best models: Random Forest and GAM^e^)	Not performed
Heikal et al (2024) [23]	ICU^f^ CatBoost: 0.974; Ward CatBoost: 0.910	Not reported
Holler et al (2025) [24]	0.77‐0.79	0.64‐0.75
Hur et al (2021) [25]	0.919 (XGBoost, best-performing model)	0.721 (Random Forest, best-performing model)
Jauk et al (2020) [26]	0.855 (prospective evaluation)	Not performed
Jauk et al (2022) [27]	Not reported	0.924‐0.931 (retrained models on external cohort)
Jauk et al (2024) [28]	0.883 (95% CI 0.852‐0.915)	Not performed
Jung et al (2022) [29]	0.80 (95% CI 0.77‐0.84)	0.82 (95% CI 0.80‐0.83)
Li et al (2024) [30]	0.92 (full feature set); 0.86 (selected feature set)	Not performed
Liu et al (2022) [31]	0.952 (combined model, 6-hour prediction window)	Not performed
Lucini et al (2023) [32]	0.909 (0‐12 hours); 0.895 (12‐24 hours)	Not performed
Matsumoto et al (2023) [33]	0.85 (cross-validation, derivation cohort)	0.86‐0.90 (XGBoost); 0.86‐0.89 (LASSO^g^); 0.84‐0.88 (logistic regression)
Moon et al (2018) [34]	0.89 (training), 0.90 (test set)	0.72
Mueller et al (2023) [35]	0.839 (GBM^h^, best-performing model)	Not performed
Pagali et al (2022) [36]	0.80 (modified MDP model)	Not performed
Reeve et al (2025) [37]	Not reported	0.77 (95% CI 0.72‐0.82)
Rudolph et al (2016) [38]	0.81 (retrospective C-statistic; 95% CI 0.80‐0.82)	0.69 (prospective C-statistic; 95% CI 0.61‐0.77)
Sheikhalishahi et al (2023) [39]	0.69‐0.81 (scenario-dependent)	Not applicable
Sun et al (2021) [40]	0.82 (admission-time model)	Up to 0.95 (discharge model; site-averaged)
Sun et al (2022) [41]	0.81‐0.85 (hospital-dependent; admission or discharge models)	Approximately 8 percentage points decrease when applied cross-hospital
Wong et al (2018) [42]	0.855 (GBM, test set)	Not performed

a AUROC: area under the receiver operating characteristic curve.

b MDP: Mayo Delirium Prediction.

c REDEEM: Risk Estimate of Delirium in Elderly Emergency Medicine.

d XGBoost: Extreme Gradient Boosting.

e GAM: Generalized Additive Model.

f ICU: intensive care unit.

g LASSO: Least Absolute Shrinkage and Selection Operator.

h GBM: Gradient Boosting Machine.

Of 29 studies, external AUROC was reported in 12 (41%) studies [15,17,19,24,25,27,29,33,34,37,38,40]. Values ranged from 0.69 in prospective validation [38] to approximately 0.95 in large multisite evaluations [40]. However, external validation datasets differed substantially in clinical setting, prevalence, and outcome definition, limiting direct ranking of models.

Precision-recall performance and threshold reporting are summarized in Table 9. Precision-recall performance was much less frequently reported than AUROC. Of 29 studies, internal precision–recall area under the curve (PR-AUC) was reported in 6 (21%) studies [19-21,30,32,39], and external PR-AUC in 2 (7%) [19,33] studies. This is an important gap because many delirium prediction settings are low-prevalence tasks where AUROC can appear favorable despite limited PPV.

**Table 9.** Precision-recall performance and threshold definition.

Study ID	Internal PR-AUC^a^	External PR-AUC	Threshold defined
Ali et al (2023) [14]	NR^b^	NR	Yes (≥14.1% risk cutoff)
Bartolacci et al (2025) [15]	NR	NR	Yes (predefined cutoffs per tool)
Bishara et al (2022) [16]	NR	NR	Yes (model-specific cutoffs)
Castro et al (2021) [17]	NR	NR	Yes (Youden-optimized cut points; eg, 0.12 and 0.15)
Ceppi et al (2023) [18]	NR	NR	Not reported
Contreras et al (2025) [19]	0.192	0.118	Yes (example threshold 0.20; performance varies by hospital; additional thresholds in supplement)
Contreras et al (2023) [20]	0.62 (95% CI 0.59‐0.64)	Not performed	Yes (Youden index)
Corradi et al (2018) [21]	0.604	Not performed	Yes (operating points chosen to maximize F_ and MCC^c^)
Davoudi et al (2017) [22]	NR	NR	Yes (Youden’s J statistic)
Heikal et al (2024) [23]	NR	NR	Yes (threshold lowered from 0.50 to 0.40)
Holler et al (2025) [24]	NR	NR	Yes (fixed threshold=0.50)
Hur et al (2021) [25]	NR	NR	No
Jauk et al (2020) [26]	NR	NR	Yes (percentile-based thresholds: top 5% very high risk; next 10% high risk)
Jauk et al (2022) [27]	NR	NR	Yes (top 15% risk classified as high or very high)
Jauk et al (2024) [28]	NR	NR	Yes (85th or 95th percentile risk cutoffs)
Jung et al (2022) [29]	NR	NR	Yes (Youden index; threshold=0.085)
Li et al (2024) [30]	0.80 (full); 0.73 (selected)	Not performed	Not reported
Liu et al (2022) [31]	NR	NR	Yes (fixed-recall analysis; example recall=0.80)
Lucini et al (2023) [32]	0.786 (0‐12 hours); 0.745 (12‐24 hours)	Not performed	Yes (threshold=0.37)
Matsumoto et al (2023) [33]	Reported (AUPRC^d^; value not specified)	Reported (AUPRC; value not specified)	Not explicitly fixed
Moon et al (2018) [34]	NR	NR	Yes (Youden-based cutoffs C1/ C2)
Mueller et al (2023) [35]	NR	NR	Yes (model-specific thresholds)
Pagali et al (2022) [36]	NR	NR	Yes (≤5%, 6%‐29%, ≥30% risk strata)
Reeve et al (2025) [37]	NR	NR	Yes (predefined PIPRA^e^ risk categories)
Rudolph et al (2016) [38]	NR	NR	Yes (risk-strata cut points)
Sheikhalishahi et al (2023) [39]	0.28‐0.45	Not applicable	No
Sun et al (2021) [40]	NR	NR	Yes (score-based alert categories)
Sun et al (2022) [41]	NR	NR	Yes (hospital-specific alert thresholds)
Wong et al (2018) [42]	NR	NR	Yes (thresholds set at 90% sensitivity and 90% specificity)

a PR-AUC: precision–recall area under the curve.

b NR: not reported.

c MCC: Matthews Correlation Coefficient.

d AUPRC: Area under the precision-recall curve.

e PIPRA: Pre-Interventional Preventive Risk Assessment.

Threshold-based metrics were difficult to compare. Although sensitivity, specificity, PPV, and NPV were often reported, thresholds were selected using different strategies, including data-driven optimization, fixed probability thresholds, and percentile-based risk strata. Few studies justified thresholds in relation to clinical resources, alert burden, or the intended intervention.

The performance synthesis therefore supports a cautious conclusion: routinely collected EHR data can discriminate delirium risk, but discrimination alone does not establish clinical usefulness. For deployment, external calibration, threshold consequences, and decision-analytic benefit are as important as AUROC.

### Validation, Calibration, and Implementation Characteristics

Validation strategies are summarized in Table 10 and show a clear gap between model development and transportability testing. Of 29 studies, 9 (31%) reported internal validation only, 8 (28%) combined internal and external validation, 2 (7%) focused on external validation only, and 2 (7%) reported temporal validation only. Of 29 studies, 3 (10%) were prospective evaluations without a distinct internal or external validation phase; 2 (7%) combined external validation with prospective evaluation; 2 (7%) combined internal, external, and prospective evaluation; and 1 (3%) reported apparent performance only.

**Table 10.** Validation strategies.

Study ID	Validation scope	Internal validation approach	External validation (type)
Ali et al (2023) [14]	External only	None or not applicable	Geographic (different hospital)
Bartolacci et al (2025) [15]	External only	None or not applicable	Geographic (independent ED^a^ cohort)
Bishara et al (2022) [16]	Internal only	Combined internal validation	None
Castro et al (2021) [17]	Internal+external	Split-sample	Geographic (multiple hospitals)
Ceppi et al (2023) [18]	Apparent only	Apparent performance only	None
Contreras et al (2025) [19]	Internal+external	Bootstrap	Geographic
Contreras et al (2023) [20]	Internal only	Combined internal validation	None
Corradi et al (2018) [21]	Internal only	Combined internal validation	None
Davoudi et al (2017) [22]	Internal only	Cross-validation	None
Heikal et al (2024) [23]	Internal+external	Combined internal validation	Geographic (cross-setting)
Holler et al (2025) [24]	Internal+external	Combined internal validation	Geographic (multihospital)
Hur et al (2021) [25]	Internal+external	Temporal internal validation	Geographic
Jauk et al (2020) [26]	Prospective only	None or not applicable	None
Jauk et al (2022) [27]	Internal+external+prospective	Cross-validation	Geographic+temporal
Jauk et al (2024) [28]	Prospective only	None or not applicable	Prospective clinical cohort
Jung et al (2022) [29]	Internal+external	Combined internal validation	Geographic (independent hospital)
Li et al (2024) [30]	Internal only	Combined internal validation	None
Liu et al(2022) [31]	Internal only	Combined internal validation	None
Lucini et al (2023) [32]	Internal only	Split-sample	None
Matsumoto et al (2023) [33]	Temporal only	Cross-validation	Temporal
Moon et al (2018) [34]	Internal+external	Split-sample	Geographic
Mueller et al (2023) [35]	Internal only	Cross-validation	None
Pagali et al (2022) [36]	Prospective only	Prospective internal validation	None
Reeve et al (2025) [37]	External+prospective	None or not applicable	Prospective clinical cohort
Rudolph et al (2016) [38]	External+prospective	Split-sample	Prospective clinical cohort
Sheikhalishahi et al (2023) [39]	Internal only	Cross-validation	None
Sun et al (2021) [40]	Internal+external	Split-sample	Geographic (multisite)
Sun et al (2022) [41]	Internal+external+prospective	Split-sample	Geographic (live or workflow)
Wong et al (2018) [42]	Temporal only	Temporal internal validation	None

a ED: emergency department.

Overall, 16 studies reported some form of external or prospective validation [14,15,17,19,23-25,27-29,33,34,37,38,40,41]. Most external validation tested transfer across hospitals, health systems, time periods, or critical care databases. Cross-setting validation, such as applying an ICU-derived model to ward patients or vice versa, was uncommon.

**Figure 2.**
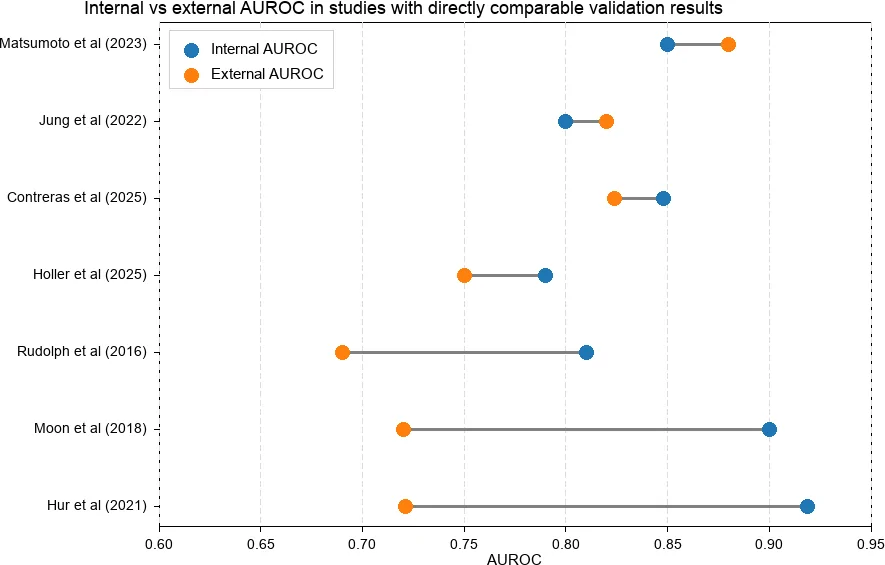
Comparison of internal and external AUROC values among studies reporting directly comparable discrimination results. AUROC: area under the receiver operating characteristic curve [19,24,25,29,33,34,38].

Among the 7 studies with directly comparable internal and external AUROC values, 5 (71%) showed lower discrimination after external validation (Figure 2). The magnitude of decline varied, indicating that performance transportability was influenced by differences in population, data capture, outcome ascertainment, and workflow context.

In this subset, mean internal AUROC was 0.845 and mean external AUROC was 0.772, corresponding to a mean change of −0.073. This descriptive comparison should not be interpreted as a pooled effect estimate, but it illustrates the risk of relying on internal performance when judging deployment readiness.

The external validation evidence was therefore mixed: several models remained discriminative outside their development data, but validation was often conducted in settings similar to the development environment. Evidence for robust transport across substantially different institutions, care pathways, and outcome assessment practices remains limited.

Calibration methods and decision curve analysis are summarized in Table 11. Of 29 studies, calibration was assessed in 15 (52%) studies [15-17,21,24-28,30,32,33,36,37,41]. Methods included calibration plots, Brier scores, Hosmer-Lemeshow tests, calibration slope or calibration-in-the-large, Platt scaling, isotonic regression, and expected calibration error. The diversity of methods, combined with incomplete reporting, made calibration difficult to compare across studies.

**Table 11.** Calibration methods and decision curve analysis.

Study ID	Calibration method	Decision curve analysis
Ali et al (2023) [14]	Not reported	No
Bartolacci et al (2025) [15]	Calibration plots, Brier score, Platt scaling, and Spiegelhalter *z* test	No
Bishara et al (2022) [16]	Calibration plots	No
Castro et al (2021) [17]	Hosmer-Lemeshow test; calibration plots	Yes
Ceppi et al (2023) [18]	Not applicable	No
Contreras et al (2025) [19]	Not reported	No
Contreras et al (2023) [20]	Not reported	No
Corradi et al (2018) [21]	Platt scaling; calibration plots	No
Davoudi et al (2017) [22]	Not reported	No
Heikal et al (2024) [23]	Not reported	No
Holler et al (2025) [24]	Calibration curves	No
Hur et al (2021) [25]	Brier score	Yes
Jauk et al (2020) [26]	Calibration plots (risk strata with confidence intervals)	No
Jauk et al (2022) [27]	Calibration plots	No
Jauk et al (2024) [28]	Calibration plots; Brier score (scaled)	No
Jung et al (2022) [29]	Not applicable	No
Li et al (2024) [30]	Expected calibration error	No
Liu et al (2022) [31]	Not reported	No
Lucini et al (2023) [32]	Isotonic regression; Brier score	No
Matsumoto et al (2023) [33]	Calibration slope, calibration intercept, and Brier score	No
Moon et al (2018) [34]	Not reported	No
Mueller et al (2023) [35]	Not reported	No
Pagali et al (2022) [36]	Calibration plots; Brier score	No
Reeve et al (2025) [37]	Calibration-in-the-large, calibration slope, and calibration plots	No
Rudolph et al (2016) [38]	Not reported	No
Sheikhalishahi et al (2023) [39]	Not reported	No
Sun et al (2021) [40]	Not reported	No
Sun et al (2022) [41]	Isotonic regression; calibration plots	Yes
Wong et al (2018) [42]	Platt scaling; calibration plots	No

Calibration reporting was often less mature than discrimination reporting. Several studies relied mainly on visual assessment, and recalibration after external validation was rare. Of 29 studies, decision curve analysis was reported in only 3 (10%) [17,25,41], leaving limited evidence about whether model-guided decisions would improve net clinical benefit.

Prospective evaluation and implementation characteristics are summarized in Table 12. Of 29 studies, 8 (28%) included prospective evaluation [26-28,34,36-38,41], and 9 (31%) reported some form of workflow integration or implementation testing [26-28,34,36-38,40,41]. These ranged from silent prospective validation to live EHR alerts, but few assessed downstream effects on clinician behavior, prevention delivery, alert burden, or patient outcomes.

**Table 12.** Prospective evaluation and implementation.

Study ID	Prospective evaluation	Implementation tested
Ali et al (2023) [14]	No	No (compared against standard VMS^a^ questions [nonintegrated comparison])
Bartolacci et al (2025) [15]	No	No
Bishara et al (2022) [16]	No	No
Castro et al (2021) [17]	No	No
Ceppi et al (2023) [18]	No	No
Contreras et al (2025) [19]	No	No
Contreras et al (2023) [20]	No	No
Corradi et al (2018) [21]	No	No
Davoudi et al (2017) [22]	No	No
Heikal et al (2024) [23]	No	No
Holler et al (2025) [24]	No	No
Hur et al (2021) [25]	No	No
Jauk et al (2020) [26]	Yes	Yes (fully embedded in hospital information system)
Jauk et al (2022) [27]	Yes	Yes (integrated into HIS^b^)
Jauk et al (2024) [28]	Yes	Yes (real-time predictions integrated into HIS; blinded to staff during study)
Jung et al (2022) [29]	No	No (web-based tool only; no real-world impact evaluation)
Li et al (2024) [30]	No	No (future integration proposed only)
Liu et al (2022) [31]	No	No
Lucini et al (2023) [32]	No	No
Matsumoto et al (2023) [33]	No	No
Moon et al (2018) [34]	Yes (after implementation)	Yes (live EHR^c^ Kardex alert)
Mueller et al (2023) [35]	No	No
Pagali et al (2022) [36]	Yes	Partial (EHR-integrated data capture; no automated alerts)
Reeve et al (2025) [37]	Yes	Yes (embedded in routine clinical workflow)
Rudolph et al (2016) [38]	Yes	Yes (EMR^d^-integrated; real-time execution approximately 8 seconds)
Sheikhalishahi et al (2023) [39]	No	No
Sun et al (2021) [40]	No	Yes (live EHR integration in 2 hospitals)
Sun et al (2022) [41]	Yes	Yes (production EHR integration)
Wong et al (2018) [42]	No	No

a VMS: Dutch safety management system (Veiligheidsmanagementsysteem).

b HIS: hospital information system.

c EHR: electronic health record.

d EMR: electronic medical record.

### Risk of Bias and Applicability

**Figure 3.**
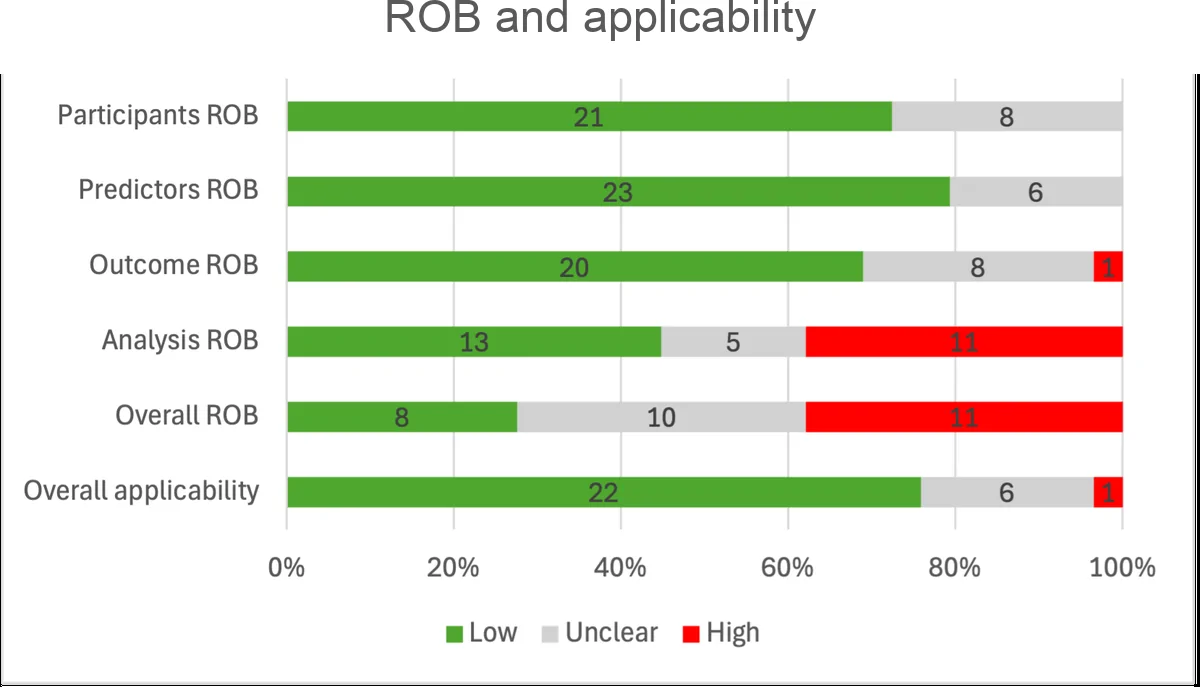
PROBAST (Prediction Model Risk of Bias Assessment Tool) stacked bar. ROB: risk of bias.

Risk of bias was assessed using PROBAST, with domain-level judgments summarized in Multimedia Appendix 4 [14-42] and overall proportions illustrated in Figure 3. Of 29 studies, overall risk of bias was judged low in 8 (28%), unclear in 10 (34%), and high in 11 (38%). The main pattern was not a lack of clinical relevance but limited methodological assurance.

Low-risk studies generally had clearer participant selection, predictor timing, outcome ascertainment, and model evaluation [15,16,21,25,32,33,36,39]. These studies provide the most reliable evidence that routinely collected data can support delirium prediction.

Studies with unclear risk of bias were usually limited by incomplete reporting rather than obvious methodological failure [17,20,22,23,29-31,35,37,42]. Common sources of uncertainty included missing data handling, calibration assessment, feature selection, and validation procedures.

High risk of bias was identified in 11 studies, most often because of analysis-domain limitations [14,18,19,24,26-28,34,38,40,41]. Recurrent issues included apparent-only performance reporting, case-control designs, inadequate handling or reporting of missing data, limited calibration assessment, and incomplete reporting of feature selection.

Within the 11 high-risk studies, inadequate handling or reporting of missing data was identified in 6 studies [26-28,34,38,41], limited or absent calibration assessment in 4 studies [14,19,34,38], apparent-only performance without validation in 1 study [18], case-control design in 2 studies [24,40], and incomplete feature selection reporting in 2 studies [14,19]. Several studies had more than 1 limitation.

These risk-of-bias findings help explain why high AUROC values should not be interpreted as readiness for practice. Models can appear accurate in development datasets while still being vulnerable to overfitting, miscalibration, missing data artifacts, or poor transportability.

Applicability concerns were more limited than risk-of-bias concerns. Most studies evaluated adult inpatient populations and used routinely collected EHR predictors, supporting broad relevance to hospital practice. However, applicability was still context-dependent for ICU-only, perioperative, emergency department, rehabilitation, COVID-19, and single health system models.

The overall quality assessment therefore supports a nuanced interpretation: the field is clinically relevant and technically active, but the evidence base is not yet consistently strong enough to support unqualified clinical deployment. No study was excluded on the basis of PROBAST assessment. Instead, risk-of-bias judgments were used to interpret how much confidence should be placed in the reported performance and implementation claims.

## Discussion

### Principal Findings

This systematic review included 29 studies developing, validating, or evaluating prediction models for in-hospital delirium using routinely collected EHR data. The principal finding is that EHR-based delirium prediction is feasible across several hospital settings, but the current evidence is fragmented across different clinical prediction tasks. The literature supports the existence of measurable risk signals in routine data; it does not yet establish that any model class is consistently ready for routine clinical deployment.

Four findings are especially important for readers. First, most models were developed retrospectively, and only a minority underwent prospective evaluation or workflow testing. Second, model performance was commonly summarized by AUROC, while calibration, precision-recall metrics, and decision-analytic evaluation were less consistently reported. Third, increased algorithmic complexity did not consistently translate into better or more transportable performance. Fourth, risk of bias was mainly driven by analysis-domain limitations rather than by lack of clinical relevance.

These findings indicate that the next stage of the field should be less focused on producing additional internally validated models and more focused on defining clinical use cases, testing transportability, calibrating models for local populations, and evaluating whether model-guided care changes decisions or outcomes.

### Interpretation in Context of Existing Literature

The heterogeneity observed in this review is consistent with previous systematic reviews of delirium prediction and broader clinical prediction modeling research [6-8]. Models differed not only in algorithm type but also in clinical setting, prediction timing, outcome ascertainment, and validation design. These differences mean that a single pooled estimate of performance would be difficult to interpret and could obscure clinically meaningful distinctions between prediction tasks.

The predominance of machine learning approaches also mirrors wider trends in hospital and critical care prediction research [43]. However, this review suggests that algorithmic sophistication is not the main bottleneck. In several studies, simpler statistical or tree-based approaches performed similarly to deep learning models, especially when evaluated under comparable conditions. Data quality, predictor timing, outcome definition, and validation context appeared at least as important as model family.

The risk-of-bias patterns also align with metaresearch showing that many published prediction models have limitations in the analysis domain, including missing data handling, overfitting, and incomplete calibration assessment [44]. Evidence that high-risk models often perform less well in external validation [45] is directly relevant here, because several delirium models showed lower discrimination when tested beyond their development data.

### Clinical Implications

Clinically, delirium prediction models are attractive because they could help target prevention, screening, and staffing resources to patients most likely to benefit. The reviewed studies show that routine EHR data contain useful risk information in ward, ICU, perioperative, and emergency care contexts. This supports continued development of delirium prediction as a component of clinical decision support.

The implementation evidence, however, remains incomplete. Few studies assessed whether predictions changed clinician behavior, reduced delirium incidence, improved patient outcomes, or avoided alert fatigue. Without these evaluations, models with favorable discrimination may still have limited value in practice, particularly if thresholds are poorly calibrated to local prevalence and available resources.

### Methodological Implications

#### Model Complexity and Performance

Across studies that directly compared modeling approaches, there was no consistent evidence that more complex models outperformed simpler alternatives. Tree-based machine learning, penalized regression, rule-based tools, and neural network approaches all achieved overlapping discrimination ranges. In several cases, models with favorable internal performance did not retain a clear advantage during external validation.

**Figure 4.**
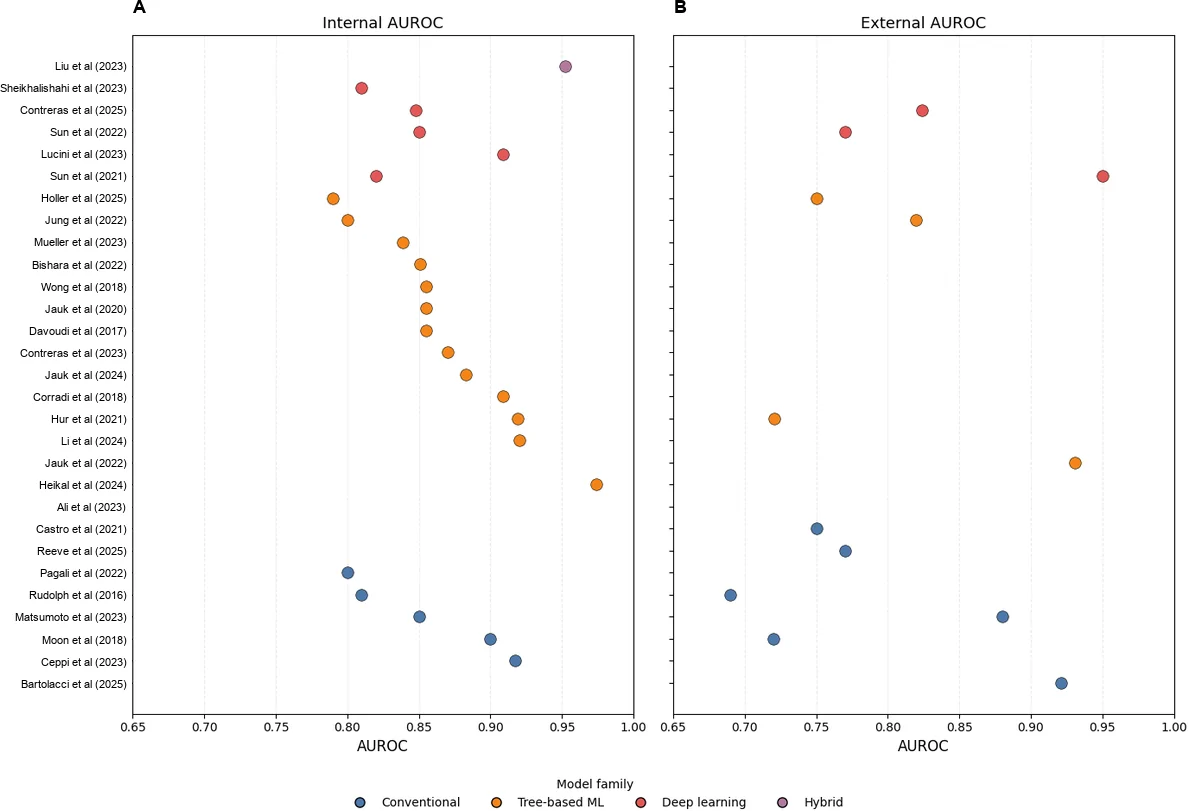
Reported AUROC by final model family across included studies: (A) internal AUROC; (B) external AUROC. AUROC: area under the receiver operating characteristic curve; ML: machine learning [14-42].

This finding does not imply that complex models are unnecessary, particularly for dynamic ICU prediction or high-dimensional time series data. Rather, it suggests that model choice should follow the clinical task, data structure, interpretability requirements, and implementation constraints. For many hospital use cases, a well-calibrated and externally validated simpler model may be more useful than a complex model with opaque behavior and limited transportability evidence. The relationship between model family and reported AUROC is shown descriptively for internal and external performance in Figures 4A and 4B, respectively.

#### Prediction Horizon and Task Comparability

Prediction horizon was one of the most important sources of heterogeneity. Admission-time models, perioperative models, rolling ICU models, and full-stay prediction models answer different clinical questions. They differ in how early an intervention can be triggered, how close predictors are to delirium onset, and how much uncertainty remains at the time of prediction.

Consequently, comparing AUROC values across prediction horizons can be misleading. A dynamic model predicting delirium in the next 12 hours may appear stronger partly because it uses proximal physiological information, whereas an admission-time model may be clinically valuable precisely because it operates before deterioration is obvious. Future studies should therefore define the intended prediction moment and intervention pathway before evaluating performance.

#### Validation and Generalizability

External validation remains the key step separating promising models from generalizable tools. Although some studies tested models outside the development dataset, validation often occurred in similar clinical settings or related health systems. Cross-setting validation and temporal validation were less common, despite being highly relevant for EHR models whose predictors and labels can change with local documentation practices.

The observed decline from internal to external AUROC in most directly comparable studies reinforces the need for conservative interpretation. Models intended for deployment should be tested across institutions, time periods, and patient groups that reflect their proposed use, and they should be recalibrated when transported to new settings.

#### Calibration and Reliability

Calibration is central to clinical reliability but was inconsistently assessed. Good discrimination indicates that a model can rank patients by risk; it does not show that predicted probabilities are accurate. For delirium prevention, inaccurate probabilities may lead to undertreatment of truly high-risk patients or excessive alerts for patients unlikely to develop delirium.

The limited use of recalibration and decision curve analysis is therefore a major evidence gap. Before deployment, models should report calibration-in-the-large, calibration slope, calibration plots, and clinically meaningful threshold analyses. Decision curve analysis or equivalent usefulness-based evaluation can help determine whether the model adds value beyond usual care or simpler screening rules.

#### Class Imbalance and Performance Metrics

Outcome prevalence varied widely, and several cohorts had low delirium prevalence. In such settings, AUROC can overstate practical usefulness because it is insensitive to the number of false positives generated at a chosen threshold. PPV and PR-AUC are especially important when the intended intervention is resource-intensive or when repeated alerts could reduce clinician trust.

The limited reporting of PR-AUC and threshold rationale therefore weakens the clinical interpretability of many studies. Future work should present threshold-specific consequences, including the number of patients flagged, false positives, false negatives, and expected resource implications at clinically plausible operating points.

#### Threshold Selection and Implementation

Threshold selection should be treated as a clinical design decision rather than a statistical afterthought. Data-driven thresholds such as Youden index may maximize a performance statistic in a development dataset, but they may not match local prevention capacity or acceptable alert burden. Fixed thresholds and percentile-based risk groups can be easier to implement, but they also require calibration to local prevalence and workflow.

For clinical deployment, threshold selection should be linked to the intended action: enhanced screening, multicomponent prevention, geriatric consultation, medication review, or ICU-specific intervention. The acceptable balance between sensitivity and specificity will differ across these use cases.

#### Use of Unstructured Data and NLP

The limited use of unstructured data is notable because delirium symptoms are often documented in narrative nursing, medical, and allied health notes. NLP may therefore improve both outcome ascertainment and predictor representation. However, free-text models raise additional challenges, including annotation burden, governance, changing documentation practices, and transportability across institutions. Future NLP-enhanced models should distinguish clearly between using language data to define the outcome and using language data as predictors. These uses have different risks for information leakage, temporal validity, and clinical implementation.

### Implementation Considerations

Several findings have direct implications for deployment. A model should not be implemented solely because it has a high AUROC. It should have an explicitly defined clinical role, evidence of calibration in the target population, threshold analyses tied to available resources, and prospective evaluation showing that predictions can be acted on without excessive alert burden.

Implementation studies should therefore evaluate not only model performance but also workflow fit, clinician response, alert fatigue, equity, prevention delivery, and patient outcomes. This is particularly important for delirium, where prediction is useful only if it leads to timely and feasible prevention or treatment.

### Limitations

Several limitations should be considered when interpreting the findings of this review. First, the quality of the included evidence base was variable, with a substantial proportion of studies judged to be at high risk of bias, primarily due to analytical limitations. Second, external validation and prospective evaluation were inconsistently performed, limiting confidence in the generalizability of reported performance.

This review also has inherent limitations. Only English-language studies were included, which may introduce language bias. The review was not prospectively registered before screening began, which may increase the risk of reporting bias. In addition, substantial heterogeneity in study design, outcome definitions, and reporting precluded formal meta-analysis. Finally, risk-of-bias assessment relied on the completeness of reporting in the original studies, which may have resulted in conservative or unclear judgments in some cases.

### Future Research Directions

Future research should move from model development toward clinically anchored validation and evaluation. The immediate priority is external and temporal validation across heterogeneous populations, with calibration assessed in ways that can support local recalibration rather than only discrimination ranking.

Implementation research should then test whether predictions change care in practice. Studies should specify the intended intervention pathway, alert threshold, resource assumptions, and monitoring plan before deployment, and should measure clinician response, alert burden, prevention delivery, equity, resource use, and patient outcomes.

Reporting should make threshold consequences easy to interpret. Future studies should present the number of patients flagged, false positives, false negatives, and expected workload at clinically plausible operating points so that health systems can judge whether a model is compatible with local capacity.

Future models may benefit from combining structured EHR variables with unstructured clinical notes, because cognitive and behavioral changes are often documented narratively. NLP-enhanced models should distinguish outcome ascertainment from predictor use, and maintain temporal separation between predictors and outcomes to avoid information leakage.

Expansion beyond delirium to broader acute mental status deterioration may be valuable only when outcomes are clearly defined, temporally valid, and clinically actionable. Across all future work, transparent reporting and alignment with TRIPOD, TRIPOD-AI, CHARMS, and PROBAST will be essential to move from technically promising models toward reliable decision-support tools.

### Conclusions

Routinely collected EHR data can support delirium prediction across hospital settings, but favorable discrimination alone does not establish clinical readiness. The evidence remains heterogeneous in outcome definition, prediction timing, prevalence, validation strategy, calibration reporting, and implementation maturity; risk of bias, particularly in the analysis domain, also remains common. More complex algorithms did not consistently improve performance. Future work should prioritize externally validated, well-calibrated, and clinically interpretable models evaluated prospectively within real workflows, with explicit attention to threshold consequences, alert burden, and patient benefit.

## Supplementary material

10.2196/91618 Multimedia Appendix 1 Full database search strategies for all electronic databases.

10.2196/91618 Multimedia Appendix 2 Detailed model development and predictor characteristics across included studies.

10.2196/91618 Multimedia Appendix 3 Model performance metrics, including discrimination and classification measures.

10.2196/91618 Multimedia Appendix 4 PROBAST (Prediction Model Risk of Bias Assessment Tool) domain-level risk of bias and applicability assessments.

10.2196/91618 Checklist 1 PRISMA checklist.

## Abbreviations

AUROC: Area under the receiver operating characteristic curve
CAM: Confusion Assessment Method
CHARMS: Checklist for Critical Appraisal and Data Extraction for Systematic Reviews of Prediction Modeling Studies
EHR: electronic health record
*ICD*: *International Classification of Diseases*
ICU: intensive care unit
NLP: natural language processing
NPV: negative predictive value
PPV: positive predictive value
PR-AUC: precision–recall area under the curve
PRISMA: Preferred Reporting Items for Systematic Reviews and Meta-Analyses
PROBAST: Prediction Model Risk of Bias Assessment Tool
TRIPOD: Transparent Reporting of a Multivariable Prediction Model for Individual Prognosis or Diagnosis
TRIPOD-AI: Transparent Reporting of a Multivariable Prediction Model for Individual Prognosis or Diagnosis–Artificial Intelligence
